# Modeling skeletal dysplasia in Hurler syndrome using patient-derived bone marrow osteoprogenitor cells

**DOI:** 10.1172/jci.insight.173449

**Published:** 2024-03-08

**Authors:** Samantha Donsante, Alice Pievani, Biagio Palmisano, Melissa Finamore, Grazia Fazio, Alessandro Corsi, Andrea Biondi, Shunji Tomatsu, Rocco Piazza, Marta Serafini, Mara Riminucci

**Affiliations:** 1 Department of Molecular Medicine, Sapienza University of Rome, Rome, Italy.; 2 Tettamanti Center, Fondazione IRCCS San Gerardo dei Tintori, Monza, Italy.; 3 Pediatrics, Fondazione IRCCS San Gerardo dei Tintori, Monza, Italy.; 4 School of Medicine and Surgery, University of Milano-Bicocca, Monza, Italy.; 5 Department of Biomedical Research, Nemours Children’s Health, Wilmington, Delaware, USA.; 6 Hematology, Fondazione IRCCS San Gerardo dei Tintori, Monza, Italy.

**Keywords:** Bone biology, Stem cells, Cartilage, Extracellular matrix, Lysosomes

## Abstract

Dysostosis multiplex is a major cause of morbidity in Hurler syndrome (mucopolysaccharidosis type IH [MPS IH], OMIM #607014) because currently available therapies have limited success in its prevention and reversion. Unfortunately, the elucidation of skeletal pathogenesis in MPS IH is limited by difficulties in obtaining bone specimens from pediatric patients and poor reproducibility in animal models. Thus, the application of experimental systems that can be used to dissect cellular and molecular mechanisms underlying the skeletal phenotype of MPS IH patients and to identify effective therapies is highly needed. Here, we adopted in vitro/in vivo systems based on patient-derived bone marrow stromal cells to generate cartilaginous pellets and bone rudiments. Interestingly, we observed that heparan sulphate accumulation compromised the remodeling of MPS IH cartilage into other skeletal tissues and other critical aspects of the endochondral ossification process. We also noticed that MPS IH hypertrophic cartilage was characterized by dysregulation of signaling pathways controlling cartilage hypertrophy and fate, extracellular matrix organization, and glycosaminoglycan metabolism. Our study demonstrates that the cartilaginous pellet–based system is a valuable tool to study MPS IH dysostosis and to develop new therapeutic approaches for this hard-to-treat aspect of the disease. Finally, our approach may be applied for modeling other genetic skeletal disorders.

## Abstract 2

A Hurler patient-derived cartilaginous pellet-based system recapitulates disease-associated skeletal alterations and highlights dysregulation of molecular pathways that may contribute to the altered phenotype.

## Graphical abstract

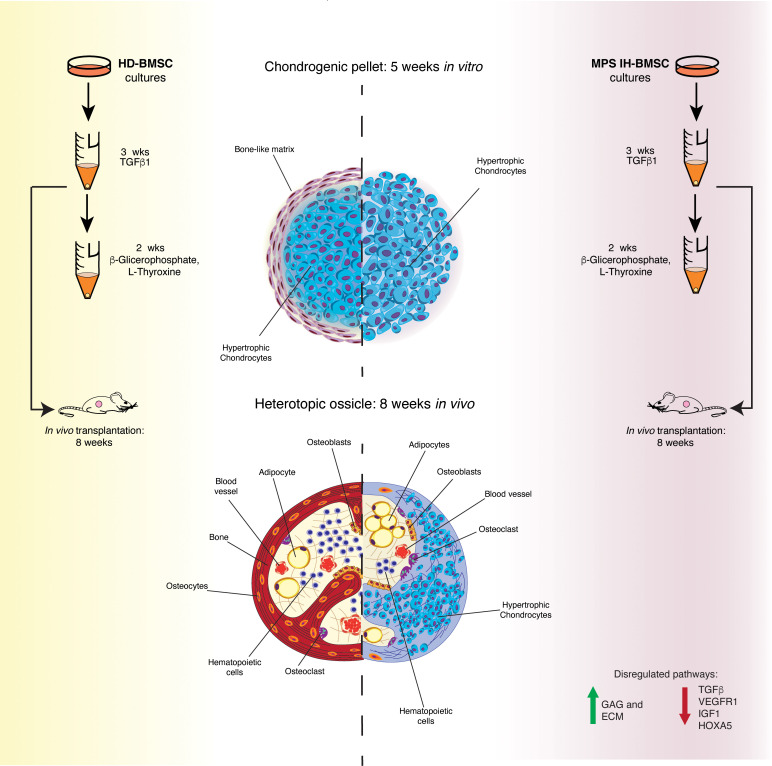

## Introduction

Mucopolysaccharidoses (MPSs) are rare, inherited lysosomal storage diseases caused by loss-of-function mutations of genes encoding glycosaminoglycan-degrading (GAG-degrading) enzymes (1, 2). MPS type I results from the lack or reduced activity of the α-L-iduronidase (IDUA) enzyme, with consequent accumulation of 2 highly abundant GAGs, heparan sulfate (HS) and dermatan sulfate (DS) (3). HS and DS storage interferes with the normal development and/or function of multiple tissues and organs, although the severity of the clinical phenotype varies according to the residual enzymatic activity. Hurler syndrome (MPS IH, OMIM #607014) is the most severe phenotype of MPS type I, reflecting absence or extremely low levels of IDUA enzymatic activity, associated with insertions/deletions, nonsense, splice variants, and missense variants in the *IDUA* gene (4). In MPS IH patients, symptoms appear shortly after birth and progress with a widespread organ involvement that, in the absence of treatment, may lead to early death. The clinical picture is dominated by progressive skeletal abnormalities with impaired musculoskeletal function, coarse facial features, hepatosplenomegaly, valvular heart disease, vision and hearing loss, upper airway obstruction, and delayed mental development with a maximal functional age of 2–4 years (5).

Dysostosis multiplex is a characterizing skeletal component of the MPS IH phenotype and a major cause of disease morbidity, as it is less likely to be prevented or resolved compared with other features by current therapies, consisting of enzyme replacement therapy and hematopoietic stem cell transplantation (6, 7). It develops in late infancy and childhood, resulting in short stature, facial dysmorphism, and several chest and limb bone deformities (8, 9). Experimental work on MPS IH transgenic mice and other spontaneously occurring dog and cat models supports the hypothesis that dysostosis multiplex may result from early developmental changes that become clinically apparent after birth (10, 11). In particular, many studies identified inappropriate retention of cartilage at sites of bone formation as a recurrent pathological feature of the disease (10, 12–14). This suggests that HS and DS accumulation during skeletal development and growth interferes with the replacement of cartilage anlage with bone and bone marrow (BM), a process that relies on endochondral ossification and, as recently shown, on the direct phenotypic switch of the cartilaginous tissue (15). However, pathological changes and mechanisms observed in MPS IH animal models could not be confirmed in human patients because performing bone biopsies at growing cartilaginous skeletal sites in pediatric patients is not advisable. Thus, there is an urgent need to develop relevant disease models that can be used to identify and study the pathophysiological mechanisms that underlie the skeletal phenotype and to adequately test new therapeutic strategies. In particular, suitable human-cell-based experimental systems that reproduce the different phases of cartilage formation as well as the different processes that ensure cartilage replacement with other skeletal tissues are highly required.

In this study, we used BM stromal cell (BMSC) populations, which include skeletal stem/progenitor cells (16, 17), isolated from pediatric MPS IH patients and performed in vitro and in vivo assays to investigate the effect of *IDUA* mutations on human cartilage features and fate. We observed that early stages of cell condensation and cartilaginous differentiation steps occurred and progressed normally in MPS IH BMSC pellet cultures. However, in the late phases of maturation, structural and molecular changes appeared and impaired the ability of MPS IH cartilage to complete its maturation in vitro and to remodel into bone and BM in vivo.

## Results

### Early stages of differentiation are preserved in MPS IH cartilage.

**Figure 1:**
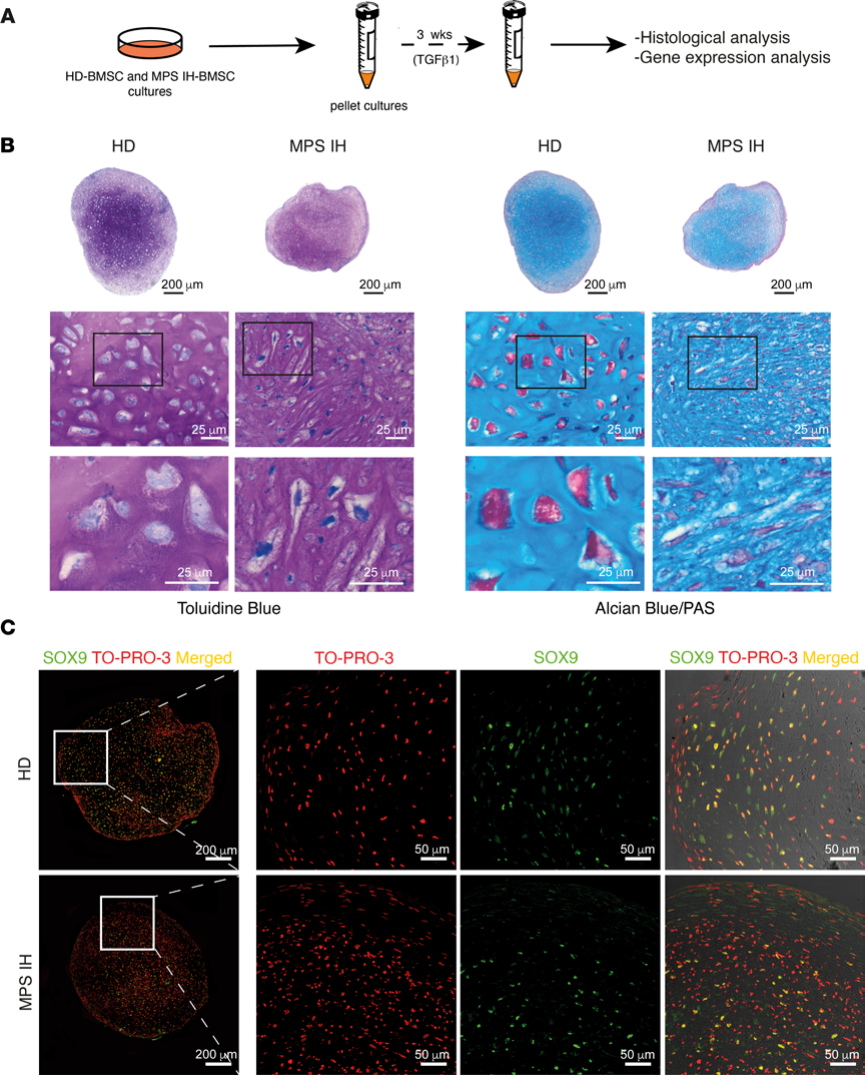
Chondrogenic differentiation of HD and MPS IH BMSCs after 3 weeks of micro-mass culture. (**A**) Schematic representation of the experimental model for cartilage differentiation. (**B**) Representative histological images of consecutive sections stained with toluidine blue and Alcian blue/PAS demonstrating similar staining for pellets generated from HD and MPS IH BMSCs. Higher-magnification images highlight the morphology of MPS IH chondrocytes, with elongated shape and indistinct lacunae. Scale bars: 25 μm. (**C**) Representative confocal images of pellet sections immunostained for SOX9 demonstrating a similar expression pattern in HD and MPS IH. Scale bars: 200 μm (left column) and 50 μm (right 3 columns).

BMSCs isolated from the BM aspirates of MPS IH patients and age-matched healthy donors (HDs) were grown to confluence and then incubated in high-density pellet cultures in the presence of TGF-β1–supplemented chondrogenic differentiation medium (CDM) (Figure 1A). After 3 weeks, MPS IH skeletal progenitor cells formed 3-dimensional cartilaginous structures similar to those formed by BMSCs isolated from HDs in terms of overall size (Supplemental Figure 1A; supplemental material available online with this article; https://doi.org/10.1172/jci.insight.173449DS1), morphology, and histochemical features, such as metachromatic staining with toluidine blue and alcianophilia (Figure 1B). Histological analysis revealed only focal morphological abnormalities for MPS IH pellets in their inner core, consisting of slender-shaped and/or vacuolated chondrocytes hosted in poorly defined lacunae (Figure 1B). Similar patterns of immunolocalization for SRY-box transcription factor 9 (SOX9) and the 2 major components of cartilage matrix, collagen type II α1 chain (COL2A1) and aggrecan (ACAN), were observed in the MPS IH and HD groups (Figure 1C and Supplemental Figure 1B). In addition, gene expression analysis confirmed the absence of significant differences between the 2 groups in the expression of *SOX9*, *COL2A1*, *ACAN*, and other cartilage genes such as *VCAN* (versican) and *HSPG2* (perlecan) (Supplemental Figure 1C).

### MPS IH cartilaginous pellets show defective remodeling into bone in vitro.

**Figure 2:**
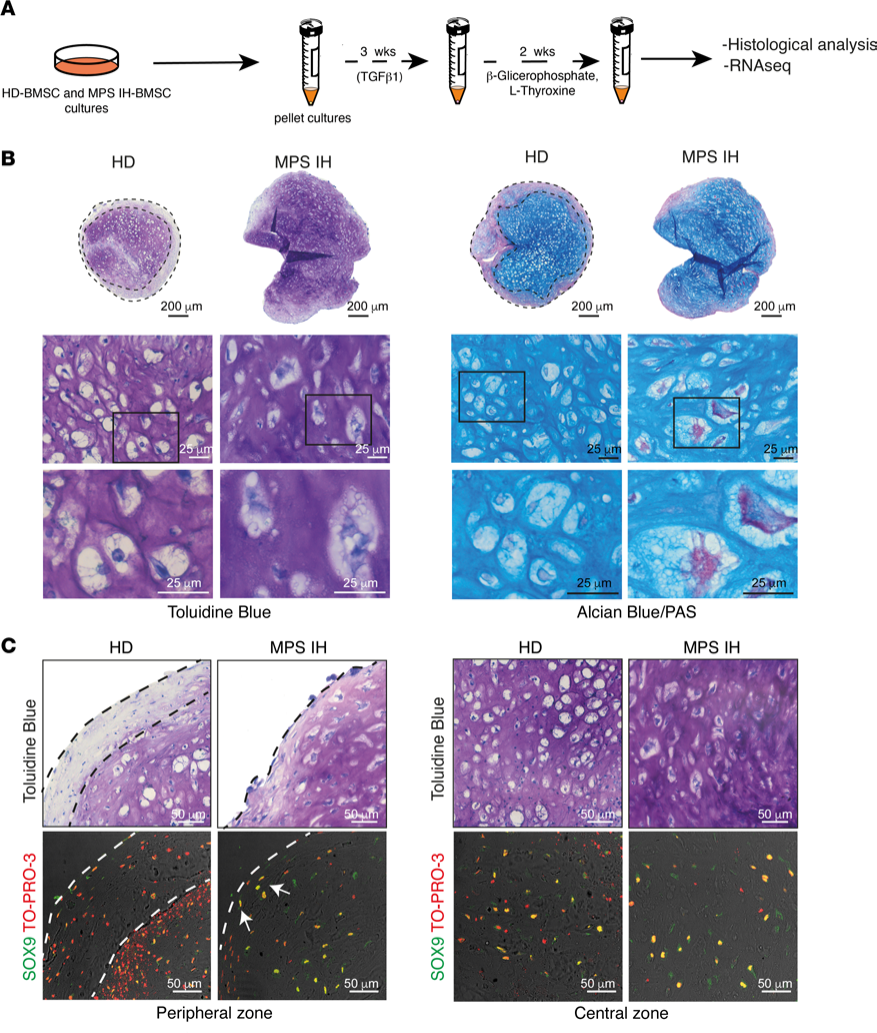
Histological and immunohistochemical analysis of 5-week HD and MPS IH pellets. (**A**) Schematic representation of the experimental model for mineralization of cartilage matrix. (**B**) Representative histological images showing the presence of the toluidine blue–negative/PAS-positive peripheral collar in HD samples (dotted lines) and its absence in MPS IH pellets. Higher-magnification bottom images show hypertrophic chondrocytes with enlarged and vacuolated cytoplasm in MPS IH compared with HD. Scale bars: 200 μm (top) and 25 μm (bottom). (**C**) SOX9 immunofluorescence demonstrating the differential expression pattern between the inner, proteoglycan-rich (toluidine blue–positive) matrix and the peripheral proteoglycan-poor (toluidine blue–negative, dotted lines) zone in HD pellets and the widespread staining in MPS IH pellets. Scale bars: 50 μm. All the toluidine blue images are derived from the same sections of the HD and MPS IH pellets represented in **B** and **C**. Therefore, different fields of the same pellet are shown in multiple panels.

To investigate the subsequent maturation of MPS IH cartilage in vitro, pellets grown in standard CDM for 3 weeks were exposed to mineralization-inductive conditions for 2 additional weeks (Figure 2A). At the end of the incubation time, 2 zones with distinct morphology and histochemical properties were recognized in HD pellets, which showed a peripheral collar composed of a dense, toluidine blue–negative and periodic acid–Schiff–positive (PAS-positive) tissue matrix and an inner region composed of large hypertrophic chondrocytes (Figure 2B). In contrast, 5-week MPS IH pellets were almost entirely composed of hypertrophic chondrocytes, often with heterogeneous size and vacuolated cytoplasm as shown by toluidine blue and Alcian blue/PAS staining (Figure 2B). At this time, SOX9 immunoreactivity showed a different distribution in HD pellets, in which it was restricted to the inner core, compared with MPS IH samples, in which it was more abundant and found throughout the whole cross-sectional area (Figure 2C).

**Figure 3:**
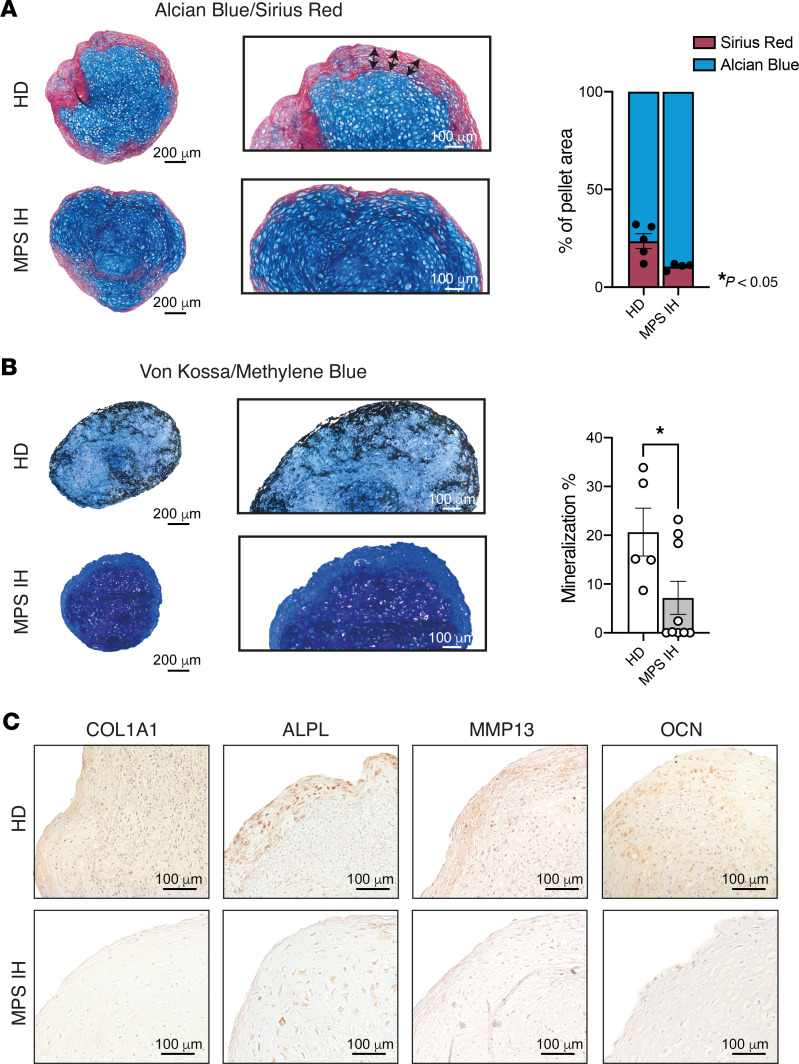
Mineralization and ossification of 5-week HD and MPS IH pellets. (**A**) Representative histological images and quantification (right graph) of pellet areas stained with Alcian blue and Sirius red, respectively, showing the COL1A1-enriched (Sirius red–stained) collar in HD samples and its reduction in MPS IH samples (pellet area stained with Sirius red, mean ± SEM from 5 HDs and 4 MPS IH): HD, 23.53% ± 3.81%; MPS IH, 10.57% ± 0.84%. *P < 0.05 by unpaired, 2-sided *t* test. (**B**) Different amounts of von Kossa–stained calcium deposition in the 2 types of pellets. Histomorphometry results (right graph) show a significant reduction in the fraction of mineralized matrix in MPS IH samples compared with HDs (pellet area stained with von Kossa, mean ± SEM from 5 HDs and 9 MPS IH): HD, 20.66% ± 4.90%; MPS IH, 7.17% ± 3.40%. *P < 0.05 by unpaired, 2-sided *t* test. Scale bars (**A** and **B**): 200 μm (left) and 100 μm (right). (**C**) Immunohistochemical staining for COL1A1, ALPL, MMP13, and OCN of 5-week HD and MPS IH pellets. Scale bars: 100 μm.

The peripheral collar of HD pellets was highly reminiscent of bone-like matrix. Indeed, double staining with Alcian blue and Sirius red performed on sections from formalin-fixed, decalcified, paraffin-embedded (FFDPE) pellets clearly distinguished the type I collagen–enriched Sirius red–stained peripheral region from the central alcianophilic region in which hypertrophic chondrocytes were dispersed (Figure 3A). This zonal pattern was poorly delineated in MPS IH samples, in which the outer Sirius red–positive border was absent or very thin and discontinuous (Figure 3A). Furthermore, von Kossa stain on undecalcified, resin-embedded pellets highlighted peripheral calcium deposits in HD but not in MPS IH pellets, in which the fraction of the mineralized extracellular matrix (ECM) was significantly reduced (Figure 3B). Consistent with these findings, an enriched immunoreactivity for COL1A1 and other proteins typically expressed during late cartilage hypertrophy and ECM mineralization, such as alkaline phosphatase (ALPL), matrix metalloproteinase 13 (MMP13), and osteocalcin (OCN), was observed in the outer layer of HD pellets but not in MPS IH samples (Figure 3C).

### Temporospatial pattern of GAG accumulation in MPS IH cartilage.

**Figure 4:**
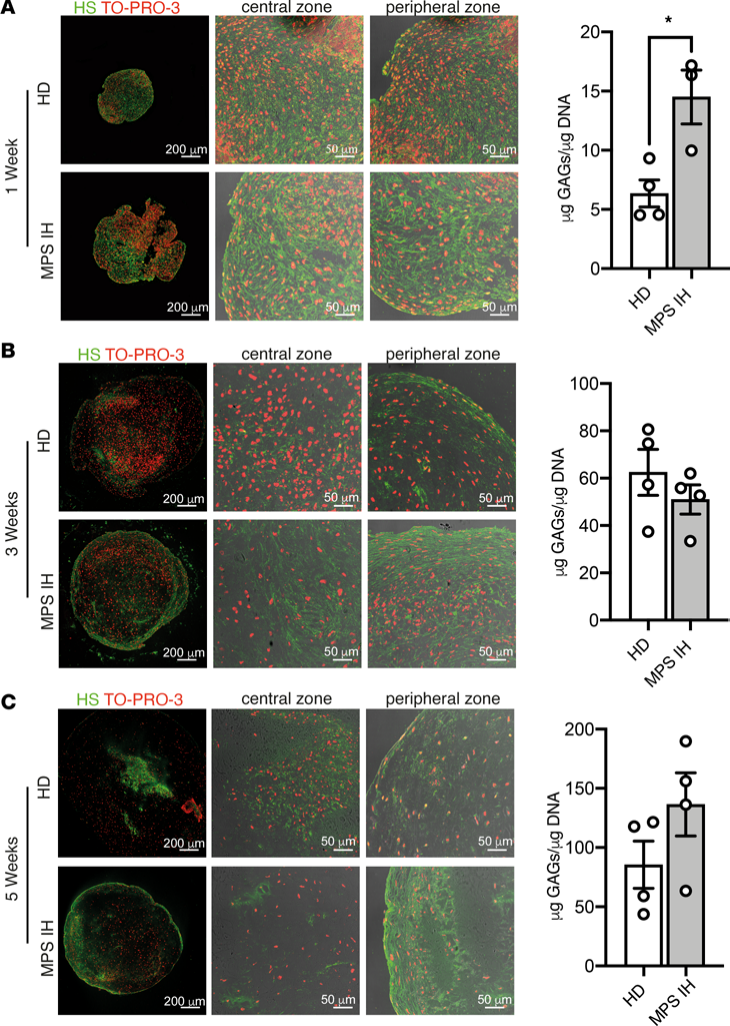
Analysis of spatial distribution of heparan sulphate and total GAG content in HD and MPS IH pellets at different stages of cartilage maturation. (**A**–**C**) Confocal images showing the expression of heparan sulphate after 1 week (**A**), 3 weeks (**B**), and 5 weeks (**C**) of chondrogenic differentiation in HD and MPS IH samples. The whole pellets and higher magnification of both central and peripheral zone are shown. The right graphs show the total GAG content expressed as μg GAG/μg DNA in each sample, at the same time points. Each dot represents 1 pellet. Data are represented as mean ± SEM. *P < 0.05 by unpaired, 2-sided *t* test. Scale bars (**A**–**C**): 200 μm (left) and 50 μm (right).

Total GAG content was assessed at different time points during in vitro chondrogenic differentiation. Comparison between the 2 experimental groups demonstrated significantly higher levels of GAGs in MPS IH compared with HD pellets at 1 week of chondrogenic differentiation (Figure 4A), thus indicating that GAG accumulation in MPS IH can be seen during cell condensation and early differentiation stages.

At 3 and 5 weeks, GAG levels were similar in HD and MPS IH pellets (Figure 4, B and C). However, immunolocalization of HS revealed a peripheral enrichment in MPS IH samples starting from week 3 as opposed to HD samples, which showed an overall even spatial distribution of the proteoglycan in the ECM at all times (Figure 4, A–C).

### Abnormal molecular profile of hypertrophic MPS IH cartilage.

To explore the molecular mechanisms involved in the delayed maturation and mineralization of the cartilage matrix of MPS IH pellets, we performed RNA-seq analysis with next-generation sequencing (NGS) on 5-week pellets. This showed 122 differentially expressed genes (DEGs), of which 87 were upregulated and 35 downregulated in MPS IH compared with HD pellets.

**Figure 5:**
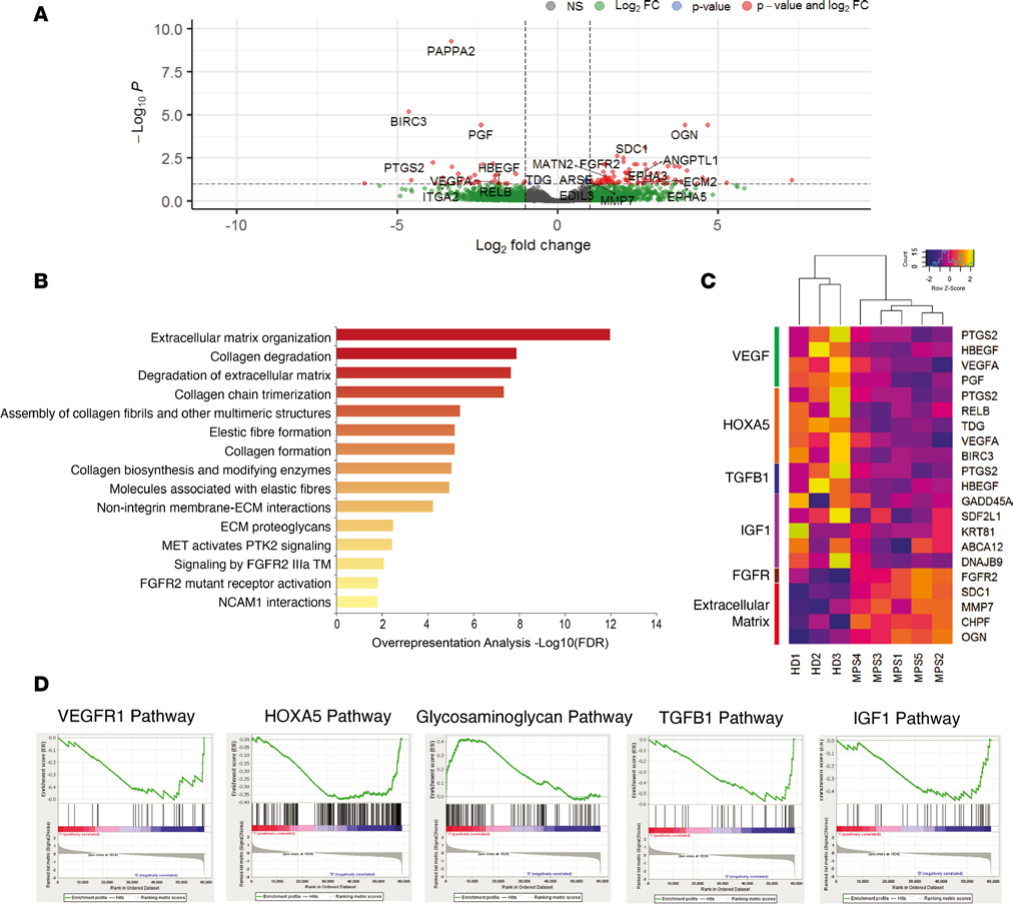
Whole-transcriptome analysis by next-generation sequencing of 5-week HD and MPS IH pellets. (**A**) Volcano plot showing the differentially expressed genes (DEGs) in MPS IH samples; each dot represents 1 gene and the log_2_(fold change) indicates the mean expression level for each gene. All genes with Benjamini-Hochberg–adjusted *P* values of less than 0.10 were considered differentially expressed. (**B**) KEGG pathway enrichment analysis showing significantly enriched pathways for DEGs in MPS IH pellets. (**C**) Heatmap representation of the top genes of different gene sets. (**D**) GSEA of transcriptome data demonstrating an underrepresentation of genes involved in VEGFR1, HOXA5, TGF-β1, and IGF1 pathways, and an overrepresentation of genes involved in the GAG pathway. GSEA gene sets with a Benjamini-Hochberg–adjusted *P* value of less than 0.25 were considered to be significantly enriched. HD, *n* = 3; MPS IH, *n* = 5.

The upregulated transcripts in MPS IH pellets included different genes involved in ECM organization and composition, such as *OGN* (osteoglycin, a small keratan sulfate proteoglycan, 15.7-fold higher), *SDC1* (syndecan-1, a transmembrane HS proteoglycan, 4.2-fold higher), *MMP7* (matrix metalloproteinase 7, a proteolytic enzyme responsible for the syndecan-1 shedding, 2.2-fold higher), *ANGPTL1* (angiopoietin-like protein 1, a gene that is known to regulate angiogenesis but it is also involved in connective tissue and cartilage development, 4.9-fold higher), and *FGFR2* (fibroblast growth factor receptor 2, a receptor involved in cartilage and bone development, 3.3-fold higher). In contrast, *PAPPA2* (pappalysin 2, a protease that cleaves insulin-like growth factor binding protein-5 [IGFBP-5] and regulates IGF signaling, 9.8-fold lower), *BIRC3* (baculoviral IAP repeat containing 3, an inhibitor of apoptosis, 24.9-fold lower), *PGF* (placental growth factor, a member of the vascular endothelial growth factor [VEGF] family, 5.3-fold lower), and *PTGS2* (prostaglandin endoperoxide synthase 2, an enzyme involved in chondrocyte hypertrophy, 14.9-fold lower) were included among the downregulated genes (Figure 5A). A Reactome pathway enrichment analysis showed an overrepresentation of genes related to GAG degradation and ECM degradation in MPS IH–derived pellets compared with controls (Figure 5B).

In line with these results, gene set enrichment analysis (GSEA) highlighted that several molecular pathways responsible for cartilage maturation and remodeling such as *VEGFR1* (VEGF receptor 1), *HOXA5* (homeobox A5), *TGFB1* (transforming growth factor β1), and *IGF1* (insulin growth factor 1) signaling were significantly downregulated in MPS IH compared with HD pellets (Figure 5, C and D). On the other hand, GAG pathway and genes related to ECM organization were upregulated in MPS IH compared with HD (Figure 5, C and D, and Supplemental Figure 2A).

To further validate our system, we compared our data with a published whole-transcriptome data set of human fibroblasts from patients with different MPS types (18). We initially generated an MPS-specific gene set by isolating all the genes consistently upregulated in MPS in the published cohort and we used it to carry out a GSEA using our gene data as ranked input. We found a statistically significant, positive enrichment (Benjamini-Hochberg–corrected *P* = 0.0069; normalized enrichment score = 1.62) between the 2, confirming that genes overexpressed in fibroblasts from different MPSs were also expressed at high levels in our data set (Supplemental Figure 2B). Subsequently, a Pearson’s correlation analysis carried out between the log_2_(fold change) of DEGs in the 2 data sets revealed a strong, positive correlation of our data, especially with MPS IH (Pearson’s *r* = 0.71) as expected, and MPS IIIC (Pearson’s *r* = 0.55).

### MPS IH cartilage shows defective remodeling into bone and BM in vivo.

**Figure 6:**
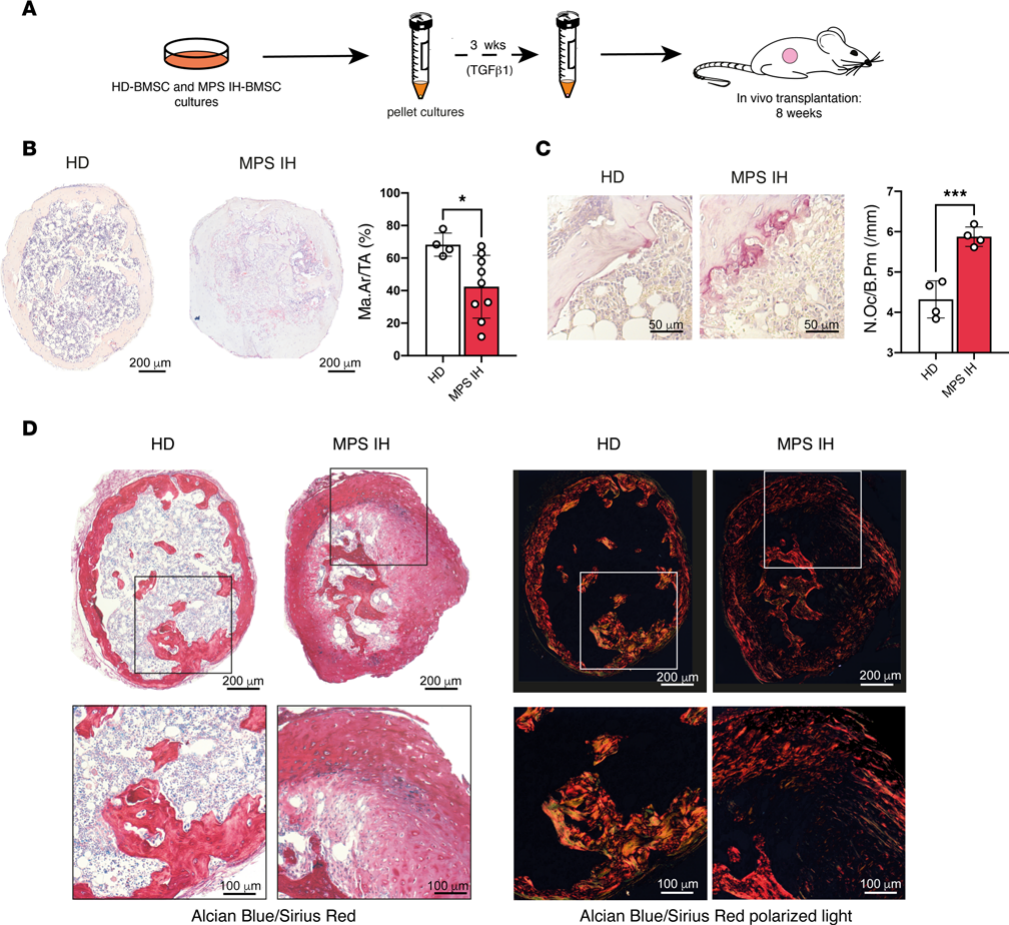
Conversion of HD and MPS IH pellets into heterotopic ossicles in vivo. (**A**) Schematic representation of the experimental model for cartilage remodeling into bone and BM in vivo. (**B**) H&E-stained sections and marrow area histomorphometry showing the reduced marrow cavity of MPS IH samples compared with HD (Ma.Ar/TA, mean ± SD from 4 HDs and 9 MPS IH): HD, 68.24% ± 7.10%; MPS IH, 42.35% ± 19.33%. *P < 0.05 by unpaired, 2-sided *t* test. Scale bars: 200 μm. (**C**) TRAP staining and histomorphometry showing the increased number of osteoclasts per bone perimeter in MPS IH ossicles compared with HD (N.Oc/B.Pm, mean ± SD from 4 HDs and 4 MPS IH): HD 4.32 ± 0.46/mm; MPS IH, 5.88 ± 0.24/mm. ***P < 0.001 by unpaired, 2-sided *t* test. Scale bars: 50 μm. (**D**) Alcian blue– and Sirius red–stained sections analyzed by transmitted (left panels) and polarized (right panels) light microscopy demonstrating retention of cartilaginous tissue (blue stain) and an overall disorganized structure in MPS IH ossicles. Scale bars: 200 μm (top) and 100 μm (higher magnification of the boxed areas, bottom).

**Figure 7:**
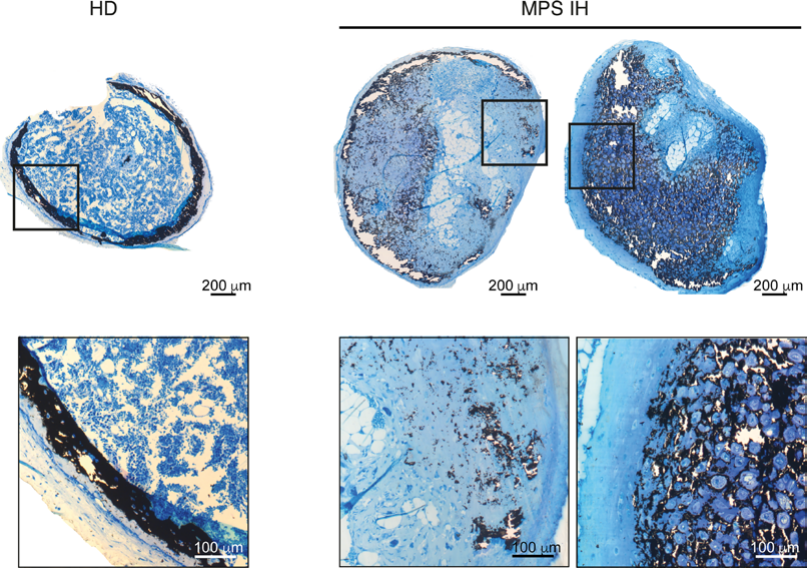
Matrix mineralization of HD and MPS IH ossicles. Von Kossa stain of undecalcified ossicles revealing the lack of a mineralized bony cortex and a variable degree of cartilage mineralization in MPS IH ossicles. Scale bars: 200 μm (top) and 100 μm (higher magnification of the boxed areas, bottom).

**Figure 8:**
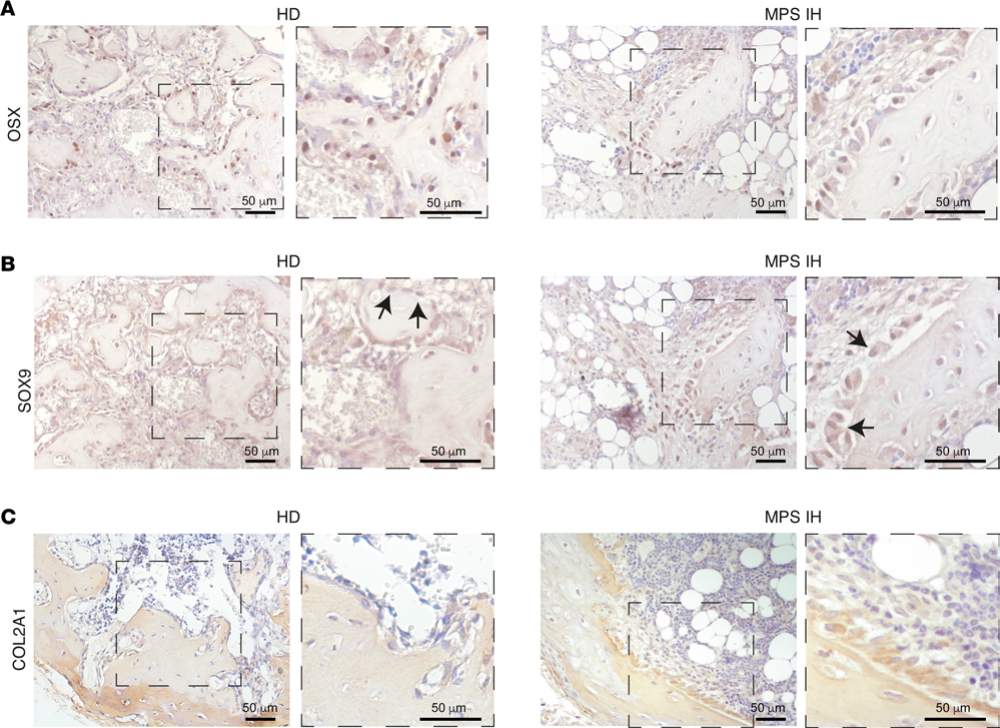
Bone-forming cells in HD and MPS IH ossicles. (**A**–**C**) Immunolocalization of OSX (**A**), SOX9 (**B**), and COL2A1 (**C**) on serial sections of HD and MPS IH ossicles showing the presence in the latter of cells with osteoblast topography and morphology coexpressing bone (OSX) and cartilage (SOX9 and COL2A1) markers. Scale bars (**A**–**C**): 50 μm.

To assess the fate of MPS IH cartilage in vivo*,* after 3 weeks of incubation in standard CDM, pellets were heterotopically transplanted into immunocompromised mice for ossicle generation (Figure 6A). Eight weeks after transplantation, hematoxylin and eosin (H&E) staining of FFDPE ossicles showed replacement of HD pellets with a thin layer of cortical-like bone and a well-structured marrow cavity hosting donor-derived adipocytes and murine hematopoiesis. In contrast, at the same time point, MPS IH ossicles were extensively composed of residual cartilage (Figure 6B). The peripheral ring of bone was not clearly defined, whereas the area of the marrow cavity was significantly smaller compared with HD and was occupied mainly by large adipocytes, with a minimal amount of murine hematopoiesis (Figure 6B and Supplemental Figure 3). Interestingly, in MPS IH ossicles, tartrate-resistant acid phosphatase–positive (TRAP-positive) osteoclasts were significantly increased compared with controls (Figure 6C). In contrast with the cortical-like bony collar observed in HD ossicles, the tissue encasing the poorly developed marrow space in MPS IH ossicles had histochemical and structural features of both bone and cartilage, as shown by double staining with Sirius red and Alcian blue (Figure 6D). Polarized light view of the same microscopic sections highlighted the absence of lamellar bone in MPS IH ossicles (Figure 6D). Moreover, von Kossa stain on undecalcified, plastic-embedded sections revealed variable levels of mineralization within the cartilaginous matrix of the MPS IH ossicles, while mineralization in the ossicles from HDs involved the cortical-like bone collar circumferentially (Figure 7). Interestingly, in MPS IH ossicles, immunolocalization of osterix (OSX) and SOX9 on adjacent tissue sections revealed cells with topographic distribution and morphological features typical of osteoblasts coexpressing both transcription factors in the nuclei (Figure 8, A and B). In the same cells, immunostaining for COL2A1 demonstrated a cytoplasmic reaction that was not observed in HD osteoblasts (Figure 8C).

### Treatment with laronidase partially reverts abnormal MPS IH cartilage phenotype in vitro.

**Figure 9:**
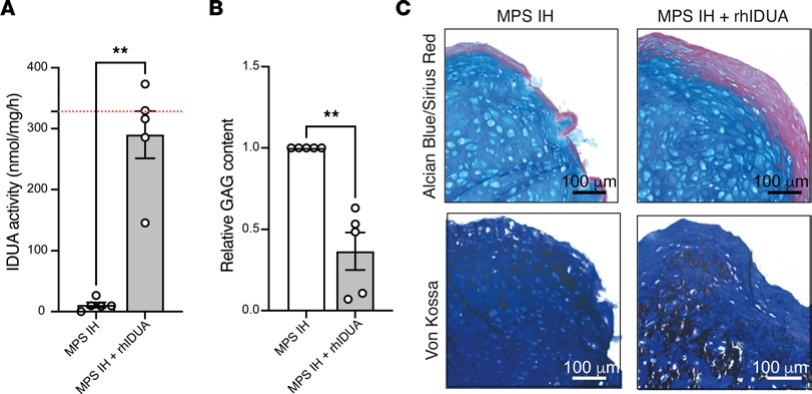
MPS IH pellets treated with laronidase. (**A**) IDUA activity in 5-week MPS IH pellets untreated or treated with laronidase (mean ± SEM from 5 MPS IH and 5 MPS IH + rhIDUA pellets): MPS IH, 10.95 ± 4.08 nmol/mg/h; MPS IH + rhIDUA, 290.1 ± 38.72 nmol/mg/h. **P *<* 0.01 by paired, 2-sided *t* test. The red dotted line represents the HD mean. (**B**) Relative GAG content in 5-week MPS IH pellets treated with laronidase compared with untreated (mean ± SEM from 5 MPS IH + rhIDUA compared with 5 untreated pellets): MPS IH + rhIDUA, 0.36 ± 0.11. **P *<* 0.01 by paired, 2-sided *t* test. (**C**) Histological images of Alcian blue– and Sirius red–stained (upper panels) and von Kossa–stained (bottom panels) sections of MPS IH pellets from the same patient untreated or treated with laronidase. Scale bars: 100 μm.

To further support our results and to start to explore the utility of the pellet system in a therapeutic context, we evaluated the effect of the treatment with the recombinant human α-L-iduronidase (laronidase) on cartilage maturation and bone-like matrix deposition in MPS IH pellets. To address this point, MPS IH BMSCs were induced for cartilaginous differentiation for 5 weeks in differentiation and mineralization culture media supplemented with 5 μg/mL laronidase. As expected, laronidase-treated MPS IH pellets showed higher IDUA activity compared with untreated MPS IH pellets, with values comparable to those of HD samples (Figure 9A). The treatment with the recombinant enzyme was able to reduce the GAG content in MPS IH pellets compared with untreated samples (Figure 9B). Importantly, histological analysis revealed a thickening of the Sirius red–stained collagenic border in MPS IH–treated pellets, indicating an increased deposition of type I collagen–enriched matrix likely reflecting an enhanced osteogenic differentiation. However, calcium deposits at this zone remained poor, despite an overall increase in mineralization with respect to untreated pellets, as showed by von Kossa stain on undecalcified sections (Figure 9C).

## Discussion

In this study, we used patient-derived models for reproducing and elucidating the cartilage pathology in MPS IH using skeletal progenitor cells. We applied these models to provide insight into the biological and transcriptional changes that are associated with endochondral ossification dysfunction in MPS IH patients, offering the first evidence to our knowledge that cartilaginous pellets and heterotopic ossicles, already employed in studies on hematological malignancies and infectious diseases (19, 20), could be used to reproduce the skeletal pathology features of an inherited disease.

The timely cartilage replacement with bone and BM at sites of endochondral ossification is required for normal skeletal development, growth, and shaping. Essential steps in the substitution process include apoptosis of hypertrophic chondrocytes, resorption of mineralized ECM, and homing of blood vessel–associated progenitor cells producing bone, marrow stroma, and marrow adipose tissue (16, 21). Accordingly, available studies on the mechanisms underlying the abnormal retention of cartilage observed in MPS IH endochondrally formed bone (12–14, 22–24) are mainly focused on the effects of GAG accumulation on this sequence of events (10, 14, 25). Recent evidence, however, demonstrates that endochondral ossification is a more complex phenomenon in which specific chondrocyte subsets escape death and undergo a phenotypic shift that leads to the direct remodeling of cartilage into other skeletal tissues (26–29). Therefore, we attempted to better understand the fate of MPS IH cartilage in the developing skeleton by recapitulating ex vivo and using human IDUA-mutated skeletal progenitor cells, the different processes, or specific aspects thereof, through which endochondral ossification unfolds.

We observed that condensation/differentiation of skeletal stem/progenitor cells into cartilaginous pellets as well as progression to hypertrophy of newly formed chondrocytes were not major targets of impaired IDUA activity, as also demonstrated in our previous work (30). However, subsequent processes leading to cartilage maturation, mineralization, and replacement with other skeletal tissues were greatly compromised/delayed in MPS IH. In particular, the ability of MPS IH cartilage to shift to a bone-like phenotype was reduced compared with normal cartilage. As previously reported (31) and as shown in this study, when cartilaginous pellets are grown in vitro under culture conditions inducing mineralization, cells in the peripheral zone assume a bone-like phenotype, expressing bone-related proteins such as COL1A1, ALPL, and OCN. Our data demonstrate that IDUA mutations interfere with this phenotypic switch, as revealed by the reduced COL1A1 deposition, matrix mineralization, and expression of bone markers detected in the outermost layer of MPS IH pellets compared with HD pellets. Impaired cartilage-to-bone transition was also observed in our in vivo model, where many cells within MPS IH ossicles seemed to retain their cartilaginous phenotype at sites of prospective bone formation.

Direct differentiation of cartilage into bone, initially suggested by ex vivo studies (32–35), was recently confirmed by lineage tracing experiments in mice as a physiological mechanism that contributes to bone formation and repair (26, 36). Thus, abnormalities of this process in MPS IH could explain, at least in part, the retention of cartilage in skeletal segments with consequent subversion of growth, maturation, and remodeling that eventually leads to bone deformities.

Interestingly, immunolabeling of 5-week MPS IH pellets showed HS enrichment and increased SOX9 expression in their peripheral layers, in contrast with HD samples in which both molecules were markedly reduced at the same time point. Previous work showed that the content of HS at the chondro-osseous junction of the growth plate must be tightly regulated for a timely progression of chondrocytes to an osteoblastic phenotype (37), and subsequent studies reported that chondrocyte-to-osteoblast conversion in the fracture callus (36) and in the growth plate (38) associates with a decrease in proteoglycan content in their pericellular matrix. Similarly, SOX9 expression was shown to contribute to chondrocyte hypertrophy and to prevent the appearance of an osteoblast phenotype in the growth plate and articular chondrocytes (38, 39). In addition, aberrant persistence of SOX9 expression was described in MPS VII mice and dogs as a mechanism contributing to the altered hypertrophic differentiation of chondrocytes (15, 40). Based on these observations, the abnormal temporospatial distribution of these 2 factors in MPS IH cartilage seems to provide a potential explanation for the defective cartilage maturation and for the reduced transition to a bone-like phenotype in vitro.

The transcriptome of 5-week MPS IH pellets further highlighted dysregulation of signaling pathways and molecules that may be implicated in cartilage development and hypertrophy and in lineage allocation of differentiated chondrocytes. For example, the RNA-seq of MPS IH pellets revealed a reduced activity of the IGF1 pathway, which it is known to be important for chondrocyte hypertrophy (41), and it is one of the few pathways so far identified as necessary for chondrocyte-to-bone transdifferentiation (42), whereas *PAPPA2* is the most downregulated gene, encoding a catabolic enzyme that positively regulates IGF bioavailability and therefore cartilaginous metabolism through collagen secretion and matrix mineralization (43). Moreover, we observed the downregulation of *HOXA5*, which inhibits *SOX9* expression, explaining at least in part the retained expression of SOX9 in the outer layer of MPS IH pellets that led to an altered hypertrophy and maturation of chondrocytes and eventually impaired their conversion into osteogenic cells (38, 44). RNA-seq also revealed abnormal levels of factors linked to GAG metabolism and ECM remodeling, consistent with data previously reported in an MPS IH mouse model (45) in which a change in ECM components, secondary to the accumulation of partially digested GAGs, was detected as a very early event in the pathogenesis of bone and joint disease (45). For example, we observed upregulation of *SDC1* and *MMP7*. SDC1 is expressed on the plasma membrane and regulates cartilage development (46), whereas MMP7 is responsible for SDC1 ectodomain shedding, resulting in the removal of bound growth factors from the cell surface. Furthermore, we found a significant upregulation of *FGFR2*. The FGF pathway plays a critical role in bone growth (47, 48) and the accumulation of abnormally sulfated HS in MPS IH may interfere with its activation by trapping FGF2 and perturbing the FGF2-FGFR-HS interactions (49, 50). Thus, the enhanced expression of the *FGFR2* gene detected in our transcriptomic analysis may reveal a compensatory mechanism to FGF seizure.

Altogether, these molecular data suggest that MPS IH cartilage is characterized by a profound derangement of multiple networks implicated in the plasticity and growth of skeletal tissues. Importantly, our results are consistent with a previously published database on human fibroblasts of different types and subtypes of MPS (18), thus suggesting a similarly disturbed regulation of gene expression hypothetically caused by common mechanisms operating in MPS as a coherent group.

Heterotopic transplantation of unmineralized pellets into immunocompromised mice allowed us to test the ability of MPS IH chondrocytes to undergo lineage reallocation in vivo and to investigate further aspects of endochondral ossification that may not be reproduced in vitro. Following transplantation, cartilaginous pellets typically generate ossicles composed of a bony cortex, sparse bone trabeculae, and a functional marrow cavity hosting murine hematopoietic cells (51, 52). In this system, cortical and trabecular bone formation relies on direct remodeling of peripheral cartilage, whereas the establishment of a BM cavity results from vascularization and resorption of the inner cartilaginous core and from the conversion of released chondrocytes into marrow stromal cells and adipocytes (51). Similar to the in vitro results demonstrating that MPS IH chondrocytes had a reduced capability to differentiate into bone-like cells, a defect/delay in cartilage-to-bone conversion was suggested also by in vivo studies. Indeed, after the canonical transplantation time, MPS IH ossicles still included a poorly developed cortical and trabecular bone and, most importantly, OSX-positive osteoblastic cells expressing SOX9 and COL2A1, suggesting a hybrid osteoblast/chondrocyte phenotype. In addition, the ossicle inner zone, normally occupied by marrow spaces, was still largely composed of a poorly vascularized cartilaginous matrix. While the reduced vascularization was consistent with the downregulation of the VEGF signaling pathway revealed by the RNA-seq analysis, the lack of proper resorption despite increased osteoclastogenesis was in agreement with previous data showing that ECM GAG storage compromises osteoclast function (14) and leads to a compensatory enhancement of their formation (14, 30).

Furthermore, our in vivo ossicles suggest that in MPS IH, the transition of chondrocytes into a skeletal cell lineage other than osteoblasts, such as marrow adipocytes and stromal cells, including hematopoietic niche cells (53), is also compromised. This type of phenotypic conversion was first observed in transplanted pellets (51) and then confirmed in other in vivo experimental models such as zebrafish (28) and mice (29). Although in this study we did not investigate in detail the marrow microenvironment in MPS IH ossicles, morphological analysis of marrow cavities clearly demonstrated enrichment in abnormally large and abundant adipocytes and a low number of murine hematopoietic cells. This suggests that once released from the ECM, MPS IH chondrocytes undergo an unbalanced differentiation toward adipocytes at the expense of stromal cells and hematopoiesis-supporting cells. Overall, these results are in agreement with previous data on the function of the BM stroma in MPS IH patients (54) and highlight the need and importance of further studies on the effect of IDUA mutations on the entire skeletal lineage.

Finally, we provide evidence that the addition of laronidase to MPS IH chondrogenic cultures led to a partial correction of the cartilage phenotype, suggesting that the model can be useful to test the efficacy of new potential therapeutic approaches for preventing or treating the skeletal changes occurring in MPS IH (55).

In conclusion, we used a patient-derived experimental model based on cartilaginous pellets and heterotopic ossicles to investigate the mechanisms underlying impaired endochondral ossification in MPS IH. Despite the limited number of BMSC populations used and the high variability in genetic background between individuals, we showed for the first time to our knowledge using this approach that IDUA mutations greatly compromise the phenotypic plasticity of cartilage as well as other critical phases of the endochondral ossification processes. Transcriptomic analysis demonstrated that this occurs through a strong perturbation of ECM composition and regulatory pathways.

To the best of our knowledge, this is the first work that aims to demonstrate that use of the pellet system can contribute to the elucidation of the mechanisms underlying a skeletal disease. This modeling becomes an even more important tool in the case of rare genetic diseases in which obtaining quality samples and in sufficient numbers is extremely difficult. Having a relevant study model that reproduces the pathological features of the skeletal disease in MPS IH is a prerequisite to design and evaluate new therapeutic strategies that can tackle the skeletal phenotype, which is difficult to prevent and correct with current treatments (55). Finally, our in vitro*–*in vivo platform may be of foremost importance to translate this approach across disease states to model other disorders that affect cartilage, bone, or both.

## Methods

### Sex as biological variable.

The sex of the HDs and the patients was not considered as a biological variable for the experiments. The SCID Beige mice used in the in vivo transplantation of cartilaginous pellets were females, as previously reported (51).

### Isolation and culture of human BMSCs.

Human BMSCs were isolated from BM of pediatric HDs (*n* = 5, 3 females and 2 males, age range 7 months to 9 years) and MPS IH patients (*n* = 8, 7 females and 1 male, age range 8 to 20 months) carrying IDUA mutations and undergoing BM collection as backup prior to allogeneic hematopoietic cell transplantation, as previously described (30). IDUA mutations of MPS IH patients are reported in Table 1. Specifically, mononuclear cells were isolated from BM aspirates using a Ficoll-Paque Plus (GE Healthcare) density gradient and seeded at a density of 2 × 10^5^ cells/cm^2^ in basal growth medium consisting of low-glucose Dulbecco’s modified Eagle’s medium (DMEM, Invitrogen), 10% fetal bovine serum (Biosera), 1% penicillin-streptomycin (Invitrogen), and 1% L-glutamine (Invitrogen). Cells were grown at 37°C in a humidified atmosphere containing 5% CO_2_. The medium was changed after 24 hours to remove nonadherent hematopoietic cells and subsequently replaced twice a week until stromal cell colonies reached 70%–80% confluence. Then, the cells were lifted with 0.25% trypsin-EDTA (Invitrogen), plated at a density of 2 × 10^3^ cells/cm^2^, and expanded in culture as multiclonal strains for a maximum of 3 passages. All BMSCs were characterized for their clonogenicity, proliferation and differentiation capacity, and surface marker expression (30).

### Generation of cartilaginous pellets.

To induce cartilaginous differentiation of MPS IH and HD BMSCs, the micro-mass culture system was used as previously described (51). Briefly, after expansion in growth medium, the cells were transferred to 15 mL polypropylene conical tubes at a density of 3 × 10^5^ cells/tube, centrifuged at 1500*g* for 5 minutes, and cultured for 3 weeks in CDM consisting of high-glucose DMEM (Gibco) supplemented with ITS Premix (BD Biosciences), 1 mM sodium pyruvate (Gibco), 50 μg/mL ascorbic acid 2-phosphate (Sigma-Aldrich), 100 nM dexamethasone (Sigma-Aldrich), 0.1 mM nonessential amino acid solution (Gibco), and 10 ng/mL TGF-β1 (Sigma-Aldrich). At the end of the incubation time, MPS IH and HD pellets were processed for paraffin embedding or used for further in vitro and in vivo assays as reported below.

### In vitro mineralization of cartilaginous matrix.

To evaluate the capacity of the cartilaginous matrix to undergo mineralization, after 3 weeks of incubation in CDM, MPS IH and HD pellets were cultured for an additional 2 weeks in mineralization medium containing 7 mM β-glycerophosphate (Sigma-Aldrich) and 50 nM thyroxine (Sigma-Aldrich) in the absence of TGF-β1, as previously reported (31). Pellets treated with the recombinant human L-IDUA were supplemented with 5 mg/mL laronidase (Aldurazyme, Sanofi) for the entire culture period.

### In vivo transplantation of cartilaginous pellets.

To evaluate the generation of complete ossicles in vivo, pellets were transplanted subcutaneously into 8- to 10-week-old immunodeficient SCID Beige female mice (CB17.Cg-*Prkdc^scid^Lyst^bg-J^*/Crl, Charles River Laboratories), as previously described (51). Briefly, after anesthesia with Zoletil 20 (5 mL/g of body weight, Virbac), 4 subcutaneous pouches were generated in the mouse back and filled with pellets (2 MPS IH and 2 HD pellets/mouse, 1 pellet/pouch). After 8 weeks, mice were sacrificed and the harvested implants were immediately fixed for histological analyses.

### Histology, histochemistry, and histomorphometry.

All samples were fixed in 4% buffered formaldehyde solution for 12 hours at 4°C. Paraffin embedding was performed according to standard procedures, with previous decalcification in 10% EDTA for the 5-week pellets and the ossicles. Paraffin sections (4 μm thick) were stained with H&E for histological analysis, with toluidine blue and Alcian blue/PAS to stain the cartilaginous matrix, and with a combination of Alcian blue (pH 2.5) and Sirius red to visualize matrix proteoglycan components and collagen fibers.

TRAP histochemistry was performed to highlight mono- and multinucleated osteoclasts using Sigma-Aldrich reagents. Briefly, 50 mg of naphthol AS-BI phosphate was dissolved in 4 mL of *N*,*N*-dimethylformamide and mixed with 4 mL of acetate buffer and 92 mL of distilled water. Then, 150 mg of tartaric acid and 30 mg of Fast Garnet were added to obtain the final working solution in which slides were incubated at 37°C for 30 minutes.

For analysis of ECM mineralization, undecalcified 5-week pellets and ossicles were embedded in glycol methacrylate. Briefly, samples were dehydrated for 15 minutes in 50% acetone, followed by 15 minutes in 95% acetone, and 15 minutes in 100% acetone; the dehydrated samples were infiltrated with solution A (10 mL of hydroxyethyl methacrylate and 2 mL of ethylene glycol monobutyl ether) at 4°C for 1 week, and with solution B (20 mL of solution A and 90 mg of benzoyl peroxide) for another week. After infiltration, the specimens were transferred to a complete embedding mixture containing 20 parts solution B and 0.5 parts solution C (15 mL of polyethylene glycol 400 and 1 mL of *N*,*N*-dimethylaniline) in embedding molds and left to polymerize for 48 hours at 4°C. All reagents were purchased from Sigma-Aldrich. Sections (3 μm thick) were stained with von Kossa and counterstained with methylene blue to distinguish mineralized and unmineralized matrix. Transmitted light and polarized light microscopy images were obtained using a Zeiss Axiophot microscope.

Histomorphometry was performed on undecalcified mineralized pellets and ossicles. Von Kossa–stained sections were used to measure the mineralization fraction of 5-week pellets, H&E- and Sirius red–stained sections were used to measure marrow area/tissue area (M.Ar/TA), and TRAP-stained sections were used to quantify the osteoclast number/bone perimeter (N.Oc/B.Pm). All histomorphometric measures were performed using ImageJ software (NIH).

### Immunohistochemistry.

For immunohistochemistry, 4-μm-thick paraffin-embedded sections were immunostained with primary polyclonal or monoclonal antibodies listed in Table 2. Before primary antibody incubation, antigen retrieval was achieved with heat-mediated or enzymatic methods, when necessary. Endogenous peroxidases were quenched by incubating the slides in 3% hydrogen peroxide (Carlo Erba) for 40 minutes. To prevent nonspecific binding of antibodies, blocking with 10% bovine serum albumin in PBS was performed. After primary antibody incubation, slides were repeatedly washed in PBS and then incubated with biotin-conjugated swine anti–rabbit IgG (P0217, Agilent) or biotin-conjugated rabbit anti–mouse IgG (P0260, Agilent) for 30 minutes at room temperature in a humidified chamber. After washing in PBS, slides were incubated with streptavidin-conjugated horseradish peroxidase (P0397, Agilent) for 30 minutes at room temperature. The peroxidase reaction was developed using a 3,3′-diaminobenzidine (DAB) substrate kit (SK-4105, Vector Laboratories).

For immunofluorescence, 4-μm-thick paraffin sections were immunostained with primary antibodies listed in Table 2. Heated antigen retrieval and blocking of nonspecific binding sites with 20% goat serum in PBS were achieved before primary antibody incubation. After washing in PBS, sections were incubated with Alexa Fluor 488–conjugated goat anti–rabbit IgG (A-11008, Invitrogen) for 30 minutes at room temperature and nuclei were stained with TO-PRO-3 (T3605, Invitrogen). Sections were coverslipped with Vectashield antifade mounting medium (H-1000, Vector Laboratories) and confocal fluorescence images were obtained using a Leica TCS SP5 confocal laser scanning microscopy system (Leica Microsystems).

### GAG quantification.

Pellets at 1, 3, and 5 weeks were snap frozen in liquid nitrogen to evaluate GAG content. Pellets were digested with 100 μg/mL papain (Sigma-Aldrich) in digestion buffer (200 mM sodium phosphate buffer pH 6.4 containing 100 mM sodium acetate, 5 mM cysteine HCl, and 10 mM EDTA-2Na). After incubation overnight at 65°C with papain, supernatants were clarified for 10 minutes at 10,000*g*. The amount of DNA in clarified supernatants of pellet homogenates was determined by a Quant-iT PicoGreen dsDNA assay kit (Invitrogen). GAG levels were measured using the Blyscan Sulfated Glycosaminoglycan colorimetric assay kit (Biocolor Ltd.) according to the manufacturer’s instructions. Chondroitin 4-sulfate was used as standard. Samples were read at 620 nm emission wavelength using a Tecan GENios microplate reader fluorometer, and GAG levels are expressed as μg GAG/μg DNA in each sample.

### IDUA activity assay.

Pellets at 5 weeks treated or not with laronidase were digested in 100 μL of 0.9% NaCl/0.4% Triton X-100/Protease Inhibitor Cocktail (P8340, Sigma-Aldrich).

Protein concentration in clarified supernatants of pellet homogenates was determined by Pierce BCA assay (Thermo Fisher Scientific). IDUA activity was measured fluorometrically using the fluorogenic substrate 4-methylumbelliferyl-α-L-iduronide (Glycosynth). Five micrograms of protein was incubated for 1 hour at 37°C with a solution of 0.1 M sodium formate buffer (pH 3.2) containing 8 mM D-saccharic acid 1,4-lactone and 0.4 mM 4-methylumbelliferyl-α-L-iduronide (Sigma Aldrich). After stopping the reaction by adding 1 mL of 0.5 M carbonate buffer (pH 10.7), fluorescence was read at 365 nm excitation and 488 nm emission wavelengths using a Tecan GENios microplate reader fluorometer.

### RNA extraction and real-time PCR.

Gene expression analysis by real-time PCR was performed on 3-week pellets. Pellets were snap frozen and homogenized using a Mikro-Dismembrator U (Gottingen). Total RNA was extracted using TRIzol reagent (Invitrogen) according to the manufacturer’s protocol. RNA was reverse transcribed with a QuantiTect Reverse Transcription Kit (Qiagen) and the real-time PCR was performed on a 7500 Fast Real-Time PCR System (Applied Biosystems) with PowerUP SYBR Green master mix (Thermo Fisher Scientific) and specific primers listed in Table 3. As reference, the housekeeping gene glyceraldehyde 3-phosphate dehydrogenase (*GAPDH*) was used. Gene expression relative to the housekeeping gene was quantified by the ΔCt method, comparing MPS IH with HD 3-week pellets.

### Library generation and RNA-seq.

RNA-seq analysis was performed on 5-week pellets by next-generation sequencing. RNA of 5-week pellets was extracted using TRIzol reagent from pellet homogenates and analyzed with a Qubit RNA HS Assay Kit (Invitrogen) and Agilent RNA 6000 Nano Kit to assess quantity and integrity before processing for library generation and sequencing.

Library preparation was performed on total RNA using the Universal RNAseq kit (Nugen, Tecan), accordingly to the manufacturer’s protocol.

RNA-seq was generated by 75 × 2 paired-end sequencing on an Illumina NextSeq 550, using a High Output v2.5 150-cycle cartridge, with an average of 40 million uniquely mapped reads per sample. Raw sequences were quality tested using FastQC (https://www.bioinformatics.babraham.ac.uk/projects/fastqc/). Subsequently, fastq reads were aligned against the reference human genome (GRCh38/hg38) using the splice-aware aligner Star (56) and the quantMode GeneCounts parameter. DESeq2 v.1.30 (57) was used to perform normalization and to calculate differential gene expression. Bam alignment files were indexed using Samtools (58). The sorted, indexed bam files, coupled with bai indexes, were then manually inspected using the Integrative Genomics Viewer (59). GSEAs were performed with GSEA software v.4.2.1 (https://www.gsea-msigdb.org/gsea/downloads.jsp) by performing 1,000 random permutations at the gene-set level.

### Statistics.

Quantitative characteristics are described as mean and standard deviation (SD) or standard error of the mean (SEM), as appropriate, and categorical variables as percentages. Pairwise comparisons were performed by means of unpaired or paired 2-sided *t* test, and the significance level was set as 0.05. All graphs and statistical analyses were produced with Prism 9.0 software (GraphPad Software). Negative binomial distribution was used to model the RNA-seq counts in order to account for RNA-seq data overdispersion. False discovery rate was controlled using the Benjamini-Hochberg procedure; the adjusted *P* value threshold for the identification of statistically significant DEGs was set to less than 0.1. Gene sets were considered to be statistically significant with of a Benjamini-Hochberg–adjusted *P* value of less than 0.25, as recommended by the GSEA team. The direction of the enrichment is indicated by normalized enrichment score, the absolute value of which was also used to prioritize the list of significant gene sets.

### Study approval.

The isolation of BMSCs from BM samples collected from pediatric MPS IH patients and HDs was approved by the Ethics Committee of San Gerardo Hospital-Monza; written informed consent from either parents or legal guardians was obtained. Experimental protocols for animal studies were approved by the Italian Ministry of Health.

### Data availability.

The data generated in this study are available within the article and the supplemental material. FASTQ files are available in the ArrayExpress-BioStudies repository (https://www.ebi.ac.uk/biostudies/arrayexpress) with accession number E-MTAB-13129. Values for all data points in graphs are reported in the Supporting Data Values file.

## Author contributions

MR, MS, AP, and SD designed the research study. MR drafted the manuscript. SD conducted in vitro and in vivo experiments and acquired histological and molecular data. AP isolated BMSCs from BM patients and conducted the experiments. BP and MF conducted experiments. RP and GF performed whole-transcriptome experiments and analyzed and interpreted the data. SD, AP, BP, AC, MR, and MS contributed to data analysis and interpretation of results. ST and AB critically revised the manuscript. The order of co–first authors was determined based on the amount of work contributed. All authors read and approved the manuscript.

## Supplementary Material

Supplemental data

Supporting data values

## Figures and Tables

**Table 1:**
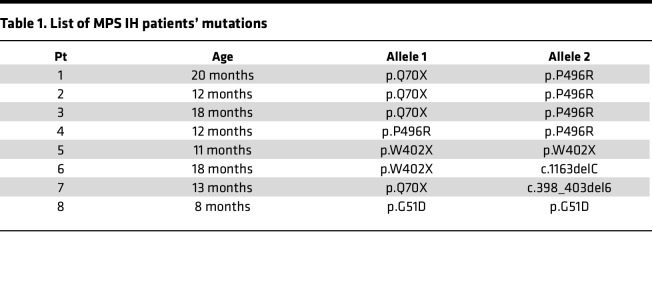
List of MPS IH patients’ mutations

**Table 2:**
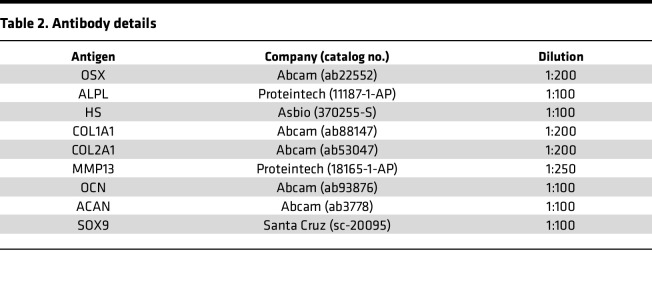
Antibody details

**Table 3:**
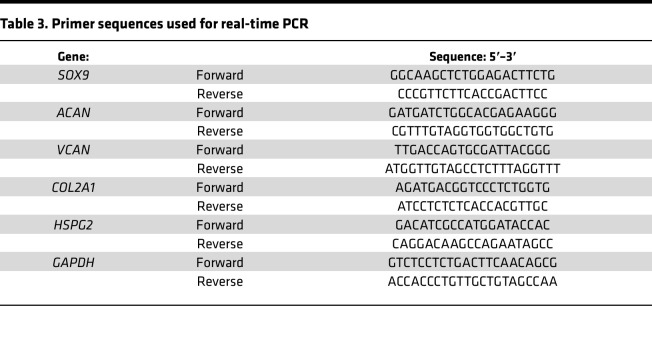
Primer sequences used for real-time PCR

## Version 1 03/08/2024

Electronic publication

## References

[3] Wraith JE, Jones S (2014). Mucopolysaccharidosis type I. Pediatr Endocrinol Rev.

[5] Muenzer J (2009). Mucopolysaccharidosis I: management and treatment guidelines. Pediatrics.

[6] Aldenhoven M (2017). Quality of life of Hurler syndrome patients after successful hematopoietic stem cell transplantation. Blood Adv.

[7] Gardin A (2023). Long term follow-up after haematopoietic stem cell transplantation for mucopolysaccharidosis type I-H: a retrospective study of 51 patients. Bone Marrow Transplant.

[8] Hampe CS (2020). Mucopolysaccharidosis type I: a review of the natural history and molecular pathology. Cells.

[9] White KK (2011). Orthopaedic aspects of mucopolysaccharidoses. Rheumatology (Oxford).

[10] Russell C (1998). Murine MPS I: insights into the pathogenesis of Hurler syndrome. Clin Genet.

[11] Rowan DJ (2013). Assessment of bone dysplasia by micro-CT and glycosaminoglycan levels in mouse models for mucopolysaccharidosis type I, IIIA, IVA, and VII. J Inherit Metab Dis.

[12] Chiaro JA (2013). Postnatal progression of bone disease in the cervical spines of mucopolysaccharidosis I dogs. Bone.

[13] Lau YK (2022). Effects of lithium administration on vertebral bone disease in mucopolysaccharidosis I dogs. Bone.

[14] Wilson S (2009). Glycosaminoglycan-mediated loss of cathepsin K collagenolytic activity in MPS I contributes to osteoclast and growth plate abnormalities. Am J Pathol.

[15] Jiang Z (2020). Failures of endochondral ossification in the mucopolysaccharidoses. Curr Osteoporos Rep.

[16] Donsante S (2021). From stem cells to bone-forming cells. Int J Mol Sci.

[17] Sacchetti B (2016). No identical “mesenchymal stem cells” at different times and sites: human committed progenitors of distinct origin and differentiation potential are incorporated as adventitial cells in microvessels. Stem Cell Reports.

[18] Gaffke L (2020). Underestimated aspect of mucopolysaccharidosis pathogenesis: global changes in cellular processes revealed by transcriptomic studies. Int J Mol Sci.

[19] Pievani A (2021). Acute myeloid leukemia shapes the bone marrow stromal niche in vivo. Haematologica.

[20] Donsante S (2023). An in vivo humanized model to study homing and sequestration of Plasmodium falciparum transmission stages in the bone marrow. Front Cell Infect Microbiol.

[21] Kronenberg HM (2003). Developmental regulation of the growth plate. Nature.

[22] Chiaro JA (2014). Effects of neonatal enzyme replacement therapy and simvastatin treatment on cervical spine disease in mucopolysaccharidosis I dogs. J Bone Miner Res.

[23] Palmucci S (2013). Imaging findings of mucopolysaccharidoses: a pictorial review. Insights Imaging.

[24] Silveri CP (1991). Hurler syndrome with special reference to histologic abnormalities of the growth plate. Clin Orthop Relat Res.

[25] Kuehn SC (2015). Impaired bone remodeling and its correction by combination therapy in a mouse model of mucopolysaccharidosis-I. Hum Mol Genet.

[26] Yang L (2014). Hypertrophic chondrocytes can become osteoblasts and osteocytes in endochondral bone formation. Proc Natl Acad Sci U S A.

[27] Zhou X (2014). Chondrocytes transdifferentiate into osteoblasts in endochondral bone during development, postnatal growth and fracture healing in mice. PLoS Genet.

[28] Giovannone D (2019). Programmed conversion of hypertrophic chondrocytes into osteoblasts and marrow adipocytes within zebrafish bones. Elife.

[29] Tan Z (2020). IRX3 and IRX5 inhibit adipogenic differentiation of hypertrophic chondrocytes and promote osteogenesis. J Bone Miner Res.

[30] Gatto F (2012). Hurler disease bone marrow stromal cells exhibit altered ability to support osteoclast formation. Stem Cells Dev.

[31] Muraglia A (2003). Formation of a chondro-osseous rudiment in micromass cultures of human bone-marrow stromal cells. J Cell Sci.

[32] Galotto M (1994). Hypertrophic chondrocytes undergo further differentiation to osteoblast-like cells and participate in the initial bone formation in developing chick embryo. J Bone Miner Res.

[33] Roach HI (1995). Osteogenic differentiation of hypertrophic chondrocytes involves asymmetric cell divisions and apoptosis. J Cell Biol.

[34] Riminucci M (1998). Vis-à-vis cells and the priming of bone formation. J Bone Miner Res.

[35] Holmbeck K (2003). MT1-MMP-dependent, apoptotic remodeling of unmineralized cartilage: a critical process in skeletal growth. J Cell Biol.

[36] Hu DP (2017). Cartilage to bone transformation during fracture healing is coordinated by the invading vasculature and induction of the core pluripotency genes. Development.

[37] Brown AJ (2008). Heparanase expression and activity influences chondrogenic and osteogenic processes during endochondral bone formation. Bone.

[38] Haseeb A (2021). SOX9 keeps growth plates and articular cartilage healthy by inhibiting chondrocyte dedifferentiation/osteoblastic redifferentiation. Proc Natl Acad Sci U S A.

[39] Dy P (2012). Sox9 directs hypertrophic maturation and blocks osteoblast differentiation of growth plate chondrocytes. Dev Cell.

[40] Peck SH (2015). Delayed hypertrophic differentiation of epiphyseal chondrocytes contributes to failed secondary ossification in mucopolysaccharidosis VII dogs. Mol Genet Metab.

[41] Wang J (1999). Igf1 promotes longitudinal bone growth by insulin-like actions augmenting chondrocyte hypertrophy. FASEB J.

[42] Aghajanian P, Mohan S (2018). The art of building bone: emerging role of chondrocyte-to-osteoblast transdifferentiation in endochondral ossification. Bone Res.

[43] Chen Y (2019). Abnormal expression of Pappa2 gene may indirectly affect mouse hip development through the IGF signaling pathway. Endocrine.

[44] Chen JW (2013). Hoxa-5 acts in segmented somites to regulate cervical vertebral morphology. Mech Dev.

[45] Heppner JM (2015). Extracellular matrix disruption is an early event in the pathogenesis of skeletal disease in mucopolysaccharidosis I. Mol Genet Metab.

[46] Salminen-Mankonen H (2005). Syndecan-1 expression is upregulated in degenerating articular cartilage in a transgenic mouse model for osteoarthritis. Scand J Rheumatol.

[47] Cinque L (2015). FGF signalling regulates bone growth through autophagy. Nature.

[48] Teven CM (2014). Fibroblast growth factor (FGF) signaling in development and skeletal diseases. Genes Dis.

[49] Pan C (2005). Functional abnormalities of heparan sulfate in mucopolysaccharidosis-I are associated with defective biologic activity of FGF-2 on human multipotent progenitor cells. Blood.

[50] Bellesso S (2018). FGF signaling deregulation is associated with early developmental skeletal defects in animal models for mucopolysaccharidosis type II (MPSII). Hum Mol Genet.

[51] Serafini M (2014). Establishment of bone marrow and hematopoietic niches in vivo by reversion of chondrocyte differentiation of human bone marrow stromal cells. Stem Cell Res.

[52] Pievani A (2017). Human umbilical cord blood-borne fibroblasts contain marrow niche precursors that form a bone/marrow organoid in vivo. Development.

[53] Sacchetti B (2007). Self-renewing osteoprogenitors in bone marrow sinusoids can organize a hematopoietic microenvironment. Cell.

[54] Baxter MA (2005). Marrow stromal cells from patients affected by MPS I differentially support haematopoietic progenitor cell development. J Inherit Metab Dis.

[55] De Ponti G (2022). MPSI manifestations and treatment outcome: skeletal focus. Int J Mol Sci.

[56] Dobin A (2013). STAR: ultrafast universal RNA-seq aligner. Bioinformatics.

[57] Love MI (2014). Moderated estimation of fold change and dispersion for RNA-seq data with DESeq2. Genome Biol.

[58] Danecek P (2021). Twelve years of SAMtools and BCFtools. Gigascience.

[59] Thorvaldsdóttir H (2013). Integrative genomics viewer (IGV): high-performance genomics data visualization and exploration. Brief Bioinform.

