# Supplementary material for: Modeling skeletal dysplasia in Hurler syndrome using patient-derived bone marrow osteoprogenitor cells
Source: JCI Insight. 2024 Mar 8;9(5):e173449. doi: 10.1172/jci.insight.173449 (PMC10972592; doi:10.1172/jci.insight.173449)
Supplement: Supplemental data [file jciinsight-9-173449-s137.pdf]

Supplemental Figures

Modeling skeletal dysplasia in Hurler syndrome using patient-derived bone marrow osteoprogenitor cells

Samantha Donsante [1*], Alice Pievani [2], Biagio Palmisano [1#], Melissa Finamore [2], Grazia Fazio [2], Alessandro Corsi [1], Andrea Biondi [3,4], Shunji Tomatsu [5], Rocco Piazza [4,6], Marta Serafini [2,4], Mara Riminucci [1].

[1] *Department of Molecular Medicine, Sapienza University of Rome, Rome, Italy.*
[2] *Tettamanti Center, Fondazione IRCCS San Gerardo dei Tintori, Monza, Italy.*
[3] *Pediatrics, Fondazione IRCCS San Gerardo dei Tintori, Monza, Italy.*
[4] *School of Medicine and Surgery, University of Milano-Bicocca, Monza, Italy.*
[5] *Department of Biomedical Research, Alfred I. duPont Hospital for Children, Wilmington, DE, USA*
[6] *Hematology, Fondazione IRCCS San Gerardo dei Tintori, Monza, Italy.*

** Present address: Tettamanti Center, Fondazione IRCCS San Gerardo dei Tintori, Monza, Italy.*
# Present address: Department of Radiological, Oncological and Pathological Science, Sapienza University of Rome.

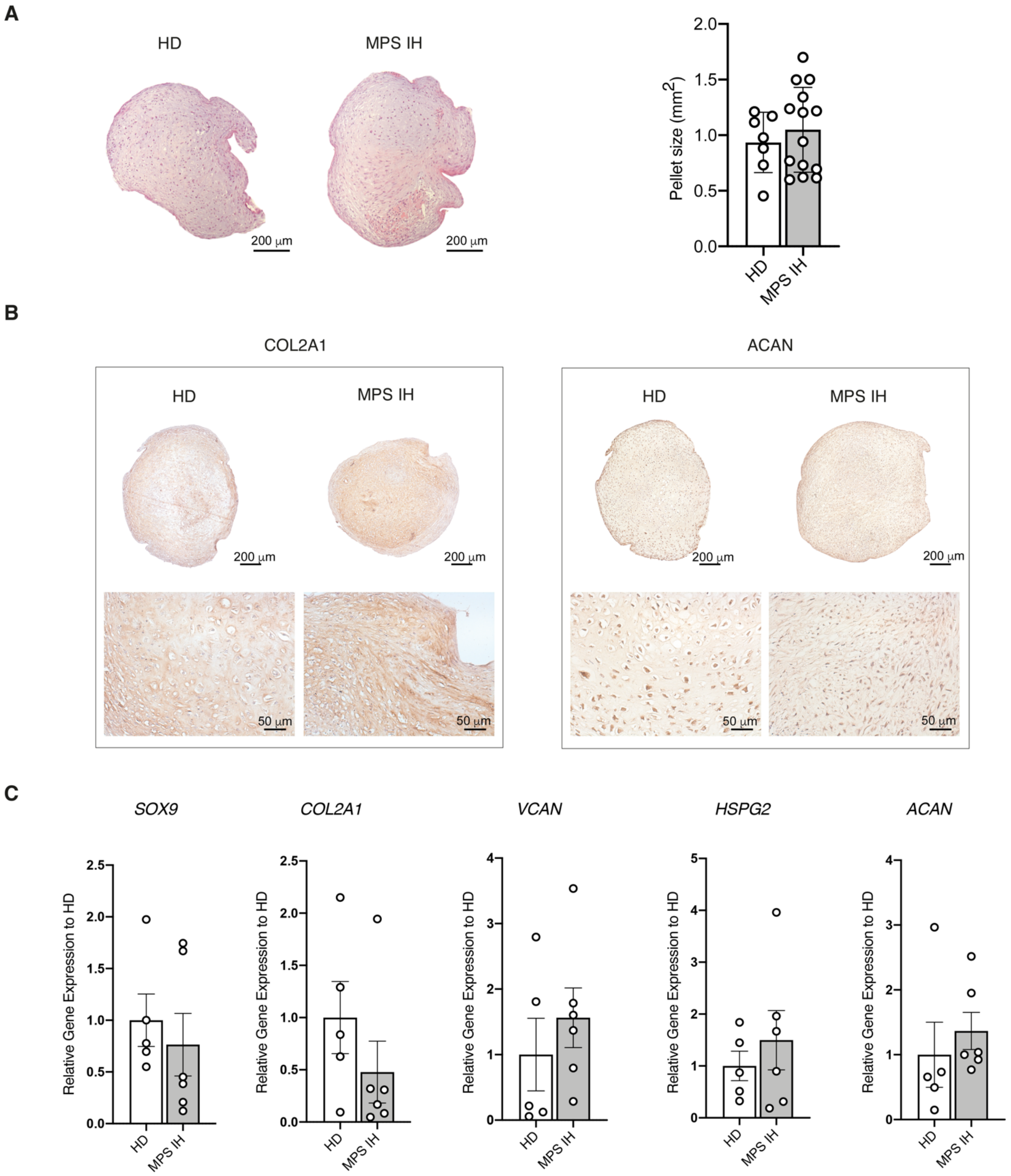

Figure S1: Histology and gene expression profile of HD and MPS IH pellets at 3 weeks.

A) Representative H&E stained histological sections and histomophometric analysis showing the similar size of HD and MPS IH pellets (mm^2, mean ± SD from 7 HD and 14 MPS IH; HD: 0.93± 0.27; MPS IH: 1.05 ± 0.38; $P > 0.05$, unpaired *t*-test). B) Immunohistochemical staining for COL2A1 and ACAN. C) Real-time PCR analysis of the cartilage markers *SOX9*, *COL2A1*, *VCAN*, *HSPG2* and *ACAN*. Results are shown as mean ± SEM (HD: n=5; MPS IH: n=6; $P > 0.05$, unpaired *t*-test).

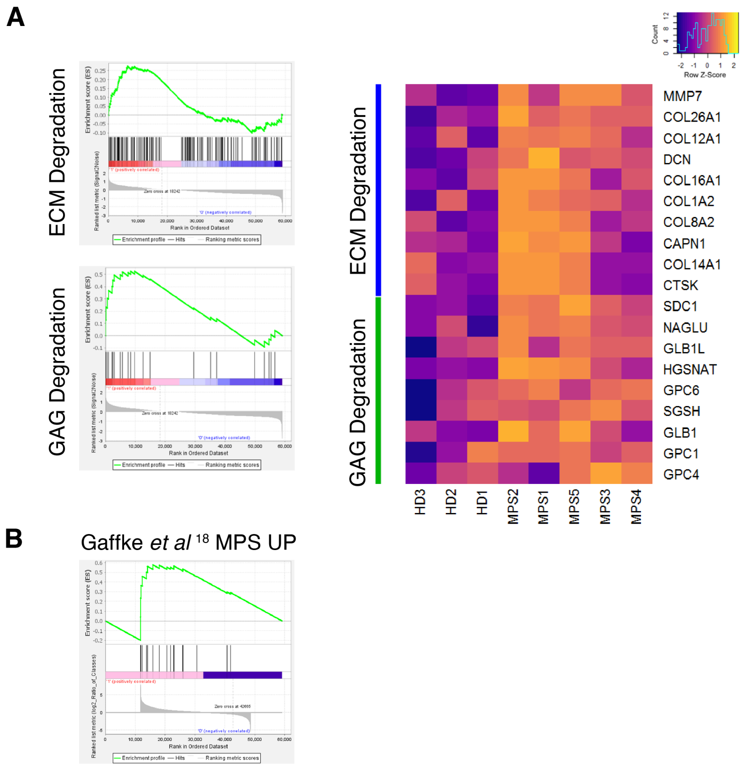

Figure S2: Gene set enrichment analysis (GSEA) of 5-week HD and MPS IH pellets.

A) GSEA analysis showing upregulation of pathways involved in ECM and GAG degradation in MPS IH pellets compared to HD samples (left panel). Heatmap representation of the top genes of each gene set (right panel). HD: n= 3; MPS IH: n=5. B) GSEA analysis showing a statistically significant positive enrichment of transcripts upregulated (UP) in the study of Gaffke et al (18) in which different MPS types were examined. Gene-sets were considered to be statistically significant in presence of a Benjamini-Hochberg adjusted p-value < 0.25.

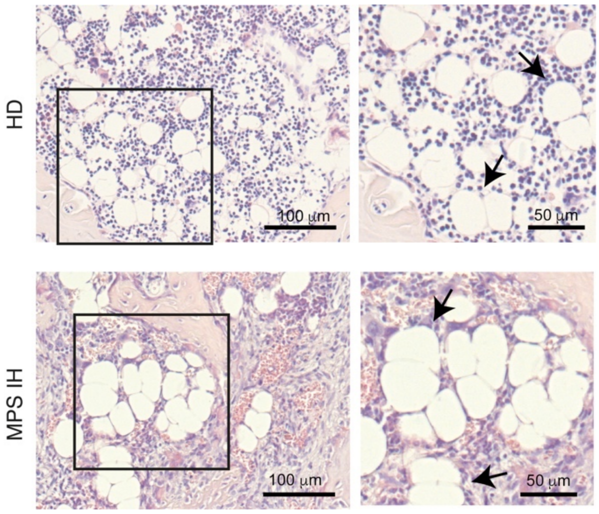

Figure S3: Histology of marrow cavity within HD and MPS IH ossicles.

Representative H&E stained histological sections showing marrow cavity with numerous adipocytes (arrows) and a reduced amount of murine hematopoiesis in MPS IH ossicles compared to HD.
